# CHA_2_DS_2_-VASc score as a mortality predictor in acute heart failure with preserved ejection fraction

**DOI:** 10.3389/fcvm.2025.1611825

**Published:** 2025-11-03

**Authors:** Olaf Kądzioła, Konrad Stępień, Alicia del Carmen Yika, Maria Kurek, Natalia Kachnic, Aleksandra Karcińska, Michael Platschek, Zuzanna Wyleciał, Karol Nowak, Aleksander Siniarski, Jadwiga Nessler

**Affiliations:** ^1^ Department of Coronary Artery Disease and Heart Failure, St. John Paul II Hospital, Kraków, Poland; ^2^ Department of Emergency Medical Services, Jagiellonian University Medical College, Kraków, Poland; ^3^ Department of Thromboembolic Disorders, Institute of Cardiology, Jagiellonian University Medical College, Kraków, Poland; ^4^ "Club 30”, Polish Cardiac Society, Warsaw, Poland; ^5^ Student Research Group at Department of Coronary Artery Disease and Heart Failure, Jagiellonian University Medical College, Kraków, Poland; ^6^ Department of Coronary Artery Disease and Heart Failure, Institute of Cardiology, Jagiellonian University Medical College, Kraków, Poland

**Keywords:** CHA₂DS₂-VASc, heart failure with preserved ejection fraction, risk stratification, mortality, acute decompensated heart failure, registry, prognosis

## Abstract

**Background:**

The mortality rate in decompensated heart failure (HF) with preserved ejection fraction (HFpEF) remains high. In recent years the prognostic role of CHA_2_DS_2_-VASc score, initially formulated for embolic risk prediction in atrial fibrillation, has been shown in other diseases including HF. We sought to analyze a long-term mortality in decompensated HFpEF patients depending on CHA_2_DS_2_-VASc score.

**Methods:**

261 (22.74%) out of 1,148 patients included in the single-center Lesser Poland Cracovian Heart Failure (LECRA-HF) Registry between 2009 and 2022 were diagnosed with decompensated HFpEF. We identified 213 (81.61%) subjects with CHA₂DS₂-VASc score ≥4 points and 48 (18.39%) < 4 points.

**Results:**

Patients with CHA₂DS₂-VASc ≥4 were older (79 vs. 64 years, *P* < 0.001), mostly females (65.3% vs. 27.1%, *P* < 0.001), and were characterized by atrial fibrillation (62.9% vs. 31.3%, *P* < 0.001), prior myocardial infarction (24.4% vs. 6.3%, *P* = 0.005), percutaneous coronary intervention (23.0% vs. 4.2%, *P* = 0.003) and coronary artery bypass surgery (11.3% vs. 2.1%, *P* = 0.049) compared to CHA_2_DS_2_-VASc <4 cohort. Lower baseline GFR (by 26.7%, *P* < 0.001), potassium (by 4.4%, *P* = 0.02), hemoglobin (by 10.3%, *P* < 0.001), as well as hematocrit (by 8.1%, *P* = 0.003) were noted in CHA_2_DS_2_-VASc ≥4 patients. In a long-term follow-up (median 4.3 years), overall mortality was significantly higher in CHA_2_DS_2_-VASc ≥4 group (*P* = 0.005) and CHA_2_DS_2_-VASc ≥4 was its independent predictor (HR 3.54, 95% confidence interval 1.68–7.49). In a multivariable Cox regression analysis, each one-point increase in CHA_2_DS_2_-VASc score raised all-cause mortality risk by 32%.

**Conclusions:**

As has been shown for the first time CHA_2_DS_2_-VASc score was an independent prognostic parameter in decompensated HFpEF.

## 1 Introduction

In accordance with the current data heart failure (HF) affects more than 64 million people worldwide (1). Approximately 50% of them suffer from HF with preserved ejection fraction (HFpEF) (2–5). The prevalence of HFpEF still increases due to better diagnostic methods, aging of the population, multimorbidity and improved survival with co-morbidities leading to HFpEF in individual patients (4, 6–9). The approximate annual all-cause mortality in general HF population was of 8.1%. This number can widely change depending on left ventricular ejection fraction (LVEF) phenotype and is 8.8% in patients with LVEF < 50%, 7.6% in patients with LVEF 40%–50% and is lowest in patients with LVEF > 50%–6.3% (10). Other study showed that the annual all-cause mortality in HFpEF reached 15% or even more in elderly patients (6). In contrast, patients with acute decompensated HF had worse clinical outcomes, reaching 35%–45% compared to 10%–20% in chronic HF (11).

To date, few well-validated scales have been developed to assess the mortality risk in HFpEF. As has been shown the MAGGIC, MEESSI-AHF, EHMRG scores could be useful to assess the risk of various clinical endpoints in HFpEF population (12–15). The CHA_2_DS_2_-VASc is a recognized worldwide, and widely recommended practical scale originally formulated for the annual thromboembolic event risk estimation and decision-making for anticoagulant treatment initiation in patients with non-valvular atrial fibrillation (AF) (15–18). However, there are plenty of studies demonstrating its usefulness of this score in assessing the risk of other clinical endpoints regardless of the AF presence. This score was also evaluated in patients with chronic obstructive pulmonary disease (COPD) (19), chronic kidney disease (17). Recently, the CHA_2_DS_2_-VASc score has also been investigated in the setting of acute coronary syndromes. In a cohort of patients with ST-segment elevation myocardial infarction (STEMI) treated with primary percutaneous coronary intervention, a score greater than two points independently predicted new-onset atrial fibrillation and hemodynamic complications such as cardiogenic shock or asystole (20, 21). In another study, patients with STEMI and a CHA_2_DS_2_-VASc ≥ 4 exhibited significantly higher in-hospital, 12-month and long-term mortality, with areas under the ROC curve of 0.88, 0.82 and 0.79, respectively (22). These findings highlight that the CHA₂DS₂-VASc score may aid risk stratification across a broad spectrum of cardiovascular conditions beyond its original thromboembolic focus.

There are also promising data regarding the use of this scale in HF, especially HF with reduced ejection fraction (HFrEF) (18, 23). Noteworthy, hypertension, older age, diabetes mellitus (DM) and coronary artery disease included in CHA_2_DS_2_-VASc score are also the significant risk factors for HFpEF (3, 4, 6, 8, 16, 24). However, the current data regarding the application of this scale in HFpEF are scarce (12, 25, 26), HFpEF remains an under-recognized entity with increasing prevalence and few evidence-based therapeutic options. Its heterogeneity and frequent comorbidities make diagnosis challenging and only limited treatments are currently available (27). This context underscores the importance of simple, inexpensive and non-invasive risk scores such as CHA_2_DS_2_-VASc, which could facilitate closer follow-up and early intervention in HFpEF patients.

To the best of our knowledge, no studies have evaluated the CHA_2_DS_2_-VASc score for risk stratification of long-term all-cause mortality among patients hospitalized with acute decompensated HFpEF. Therefore, based on data from the Lesser Poland Cracovian Heart Failure (LECRA-HF) Registry, we investigated the prognostic utility of the CHA₂DS₂-VASc score for long-term all-cause mortality in patients with HFpEF.

## 2 Materials and methods

### 2.1 LECRA-HF registry

The LECRA-HF Registry (NCT05746923) is a constantly updated database of patients admitted to the Department of Coronary Artery Disease and Heart Failure in St. John Paul II Hospital in Kraków, Poland, between 2009 and 2022 hospitalized due to the acute decompensation of HF (28). We obtained all-cause mortality follow-up from the Polish National Death Registry, censored April 7, 2024. The study protocol is compliant with the Declaration of Helsinki and was approved by the local Ethics Committee (Consent No. 1072.6120.349.2022). Each patient included in LECRA-HF gave the informed consent. The data in LECRA-HF will be continuously collected until 2026. The LECRA-HF Registry contains data of consecutive 1148 adult (aged 18 years or older) patients treated in tertiary clinic due to its acute decompensation (28, 29). The detailed characteristics included demographic and anthropometric data, as well as cardiovascular risk factors, comorbidities, pharmacological treatment, transthoracic echocardiography (TTE) parameters, laboratory results, all collected during the index hospitalization. Based on LECRA-HF, 261 (22.74%) were diagnosed with decompensated HFpEF.

### 2.2 HFpEF definition

HFpEF was diagnosed according to the European Society of Cardiology (ESC) guidelines in force at the time of the index hospitalization (30–33). To harmonize case ascertainment across 2009–2022, we required: (1) signs and/or symptoms of heart failure; (2) left-ventricular ejection fraction ≥50% measured by the biplane Simpson method (34); and (3) evidence of elevated filling pressures documented by natriuretic peptides and/or echocardiography, as available. Consistent with ESC guidance for the acute setting, an admission NT-proBNP ≥ 300 pg/ml was considered biochemical evidence of elevated filling pressures (32, 33). When both natriuretic peptides and echocardiography were available, both were expected to support the diagnosis; when NT-proBNP did not meet the threshold defined above or was not measured, echocardiographic evidence of structural heart disease and/or diastolic dysfunction was required in accordance with ESC criteria current at the time of the index hospitalization (30–34).

### 2.3 Assessments and variable definitions

HF symptoms were classified by the New York Heart Association class (NYHA), while laboratory tests and TTE were performed by a qualified medical staff according to the current standards (34). N-terminal pro-B-type natriuretic peptide (NT-proBNP) level was measured using standard methods (35). Renal function was established based on the glomerular filtration rate (GFR) < 30 ml/min using the Cockroft-Gault formula (35). LVEF and left atrial volume were measured using the biplane Simpson method (34). COPD was diagnosed accordingly to the current criteria based on the GOLD guidelines (36). DM was defined as treatment with oral hypoglycemic drugs, insulin or both equally, as well as fasting blood glucose level >125 mg/dL (7 mmol/L) (16).

### 2.4 CHA_2_Ds_2_-VASc score

CHA_2_DS_2_-VASc score was calculated for all patients as validated for anticoagulant initiation in AF, giving 1 point for each category: congestive HF, hypertension, age 65–74 years, DM, vascular disease and sex category (female), while 2 points for age ≥75 years and for previous stroke/transient ischemic attack/thromboembolism incident (16).

### 2.5 Statistical analysis

Categorical variables were reported as numbers and percentages. Continuous variables were presented as median (first and third quartile, Q1-Q3). Shapiro–Wilk test was used to assess the normal distribution of variables. The continuous variables were compared between two groups using Student's or U-Mann Whitney tests. Categorical variables were analyzed using the *χ*^2^ test or the Fisher exact test. The associations between numerical variables were assessed by Pearson's or Spearman's rank correlation coefficient. Kaplan–Meier curves of all-cause mortality in the studied groups were prepared and compared with the log-rank test. Receiver operating characteristic (ROC) curves and the area under the curve (AUC) were computed for CHA₂DS₂-VASc, MAGGIC, EHMRG, and MEESSI-AHF using nonparametric methods. Pairwise comparisons of AUCs were performed using DeLong's test with two-sided *P*-values.

The multivariable Cox proportional hazard regression analysis was performed to investigate the relationships of CHA_2_DS_2_-VASc with mortality. Two sets of models were conducted: CHA_2_DS_2_-VASc as numerical variable and as dichotomized variable (CHA_2_DS_2_-VASc ≥4). The final models were adjusted for NYHA, COPD, renal failure, hemoglobin and maximal aortic gradient. The selection of variables for the multivariable model was based on the results in univariable models, literature and to avoid collinearity. Moreover, to avoid overfitting given the relatively small number of events, we limited the number of covariates in the multivariable model. Established prognostic markers such as NT-proBNP are highly correlated with NYHA class and renal function. Including them introduced multicollinearity and did not materially change the hazard ratios of the variables retained in the model. Therefore, for statistical and practical reasons we chose to exclude NT-proBNP and detailed LVEF categorizations from the final models. The results of Cox regression were presented as hazard ratios (HR) with 95% confidence interval (CI). The f goodness-of-fit of preformed model were assessed using Harrell's C-index. A two-sided *P*-value of less than 0.05 was considered statistically significant. All statistical analyses were performed using STATISTICA software Version 13.3 (StatSoft, Krakow, Poland) or R Core Team (2013, Vienna, Austria).

## 3 Results

**Figure 1:**
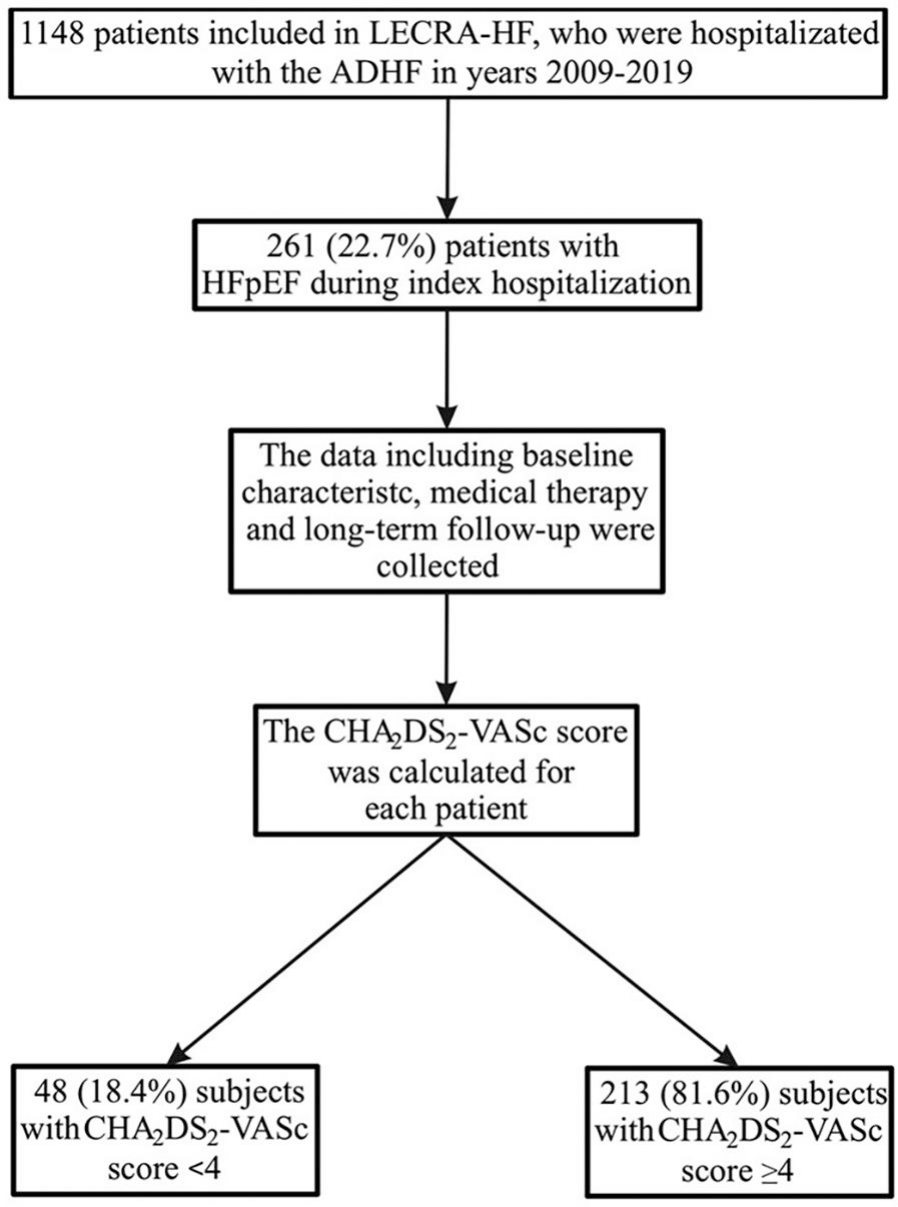
Study flowchart. ADHF, acute decompensated heart failure; HFpEF, heart failure with preserved ejection fraction.

We analysed 261 patients hospitalized for decompensated HFpEF with a median follow-up of 4.3 years. Of these, 213 patients (81.6%) had a CHA_2_DS_2_-VASc score ≥ 4 and 48 (18.4 %) had a score <4 (Table 1; Figure 1).

**Table 1:** Baseline characteristics of the studied patients.

Variable	CHA_2_DS_2_-VASc <4 (*n* = 48)	CHA_2_DS_2_-VASc ≥4 (*n* = 213)	*P*-value
Female gender, %	13 (27.1)	139 (65.3)	**<0** **.** **001**
Age, years	64 (56–69)	79 (71–84)	**<0**.**001**
Body mass index, kg/m^2^	27.5 (23.1–30.5)	29.2 (26.2–34.3)	0.09
Systolic blood pressure on admission, mmHg	130 (113–154)	139 (122–160)	0.06
Diastolic blood pressure on admission, mmHg	75 (65–89)	78 (67–90)	0.55
Heart rate, beats/min	75 (65–90)	76 (66–94)	0.41
NYHA III/IV, %	28 (58.3)	151 (70.9)	0.36
Prior myocardial infarction, %	3 (6.3)	52 (24.4)	**0**.**005**
Percutaneous coronary intervention, %	2 (4.2)	49 (23.0)	**0**.**003**
Coronary artery bypass surgery, %	1 (2.1)	24 (11.3)	**0**.**049**
Hypertension, %	25 (52.1)	200 (93.9)	**<0**.**001**
Hyperlipidemia, %	21 (43.8)	136 (63.9)	**0**.**010**
Diabetes mellitus, %	3 (6.3)	93 (43.7)	**<0**.**001**
Renal failure, %	10 (20.8)	86 (40.4)	**0**.**014**
Peripheral arterial disease, %	2 (4.2)	41 (19.2)	**0**.**011**
Atrial fibrillation, %	15 (31.3)	134 (62.9)	**<0**.**001**
Paroxysmal	5 (10.4)	37 (17.3)	**0**.**001**
Permanent	10 (20.8)	97 (45.5)	**0**.**001**
Stroke, %	1 (2.1)	19 (8.9)	0.11
History of cancer, %	3 (6.3)	35 (16.4)	0.07
COPD, %	11 (22.9)	27 (12.7)	0.07
Pacemaker, %	13 (27.1)	34 (16.0)	0.12
Coronary invasive diagnostics and treatment during hospitalization, %			
Coronary angiography	20 (41.7)	50 (23.5)	**0**.**010**
Significant coronary stenosis	1 (2.1)	14 (6.6)	0.23
Percutaneous coronary intervention	1 (2.1)	6 (2.8)	0.78
Coronary artery bypass surgery	0 (0.0)	2 (0.9)	0.50
Treatment at discharge, %			
ACEI	29 (61.4)	126 (59.2)	0.68
Beta-blocker	40 (83.3)	182 (85.4)	0.30
Mineralocorticoid receptor antagonist	14 (29.2)	84 (39.4)	0.35
Loop diuretic	27 (56.3)	165 (77.5)	**0**.**001**
Digoxin	9 (18.8)	33 (15.5)	0.89
Statin	21 (43.8)	150 (70.4)	**<0**.**001**
Direct oral anticoagulant	8 (16.7)	64 (30.0)	0.06
Vitamin K antagonist	13 (27.1)	88 (41.3)	0.07
Acetylsalicylic acid	15 (31.3)	117 (54.9)	**0**.**003**
P2Y12 inhibitor	2 (4.2)	14 (6.6)	0.53
Metformin	3 (6.3)	57 (26.8)	**0**.**002**
Insulin	4 (8.3)	68 (31.9)	**0**.**001**

ACEI, angiotensin-converting enzyme inhibitors; COPD, chronic obstructive pulmonary disease; NYHA, New York Heart Association.

Bold value indicates *P* < 0.05.

### 3.1 Baseline characteristics of the studied patients

Patients with CHA_2_DS_2_-VASc ≥4 were more often female (*P* < 0.001), older (*P* < 0.001) and more frequently had hypertension (*P* < 0.001), hyperlipidemia (*P* = 0.01), DM (*P* < 0.001), renal failure (*P* = 0.01) and peripheral arterial disease (*P* = 0.01) (Table 1). They were also more often diagnosed with atrial fibrillation, both paroxysmal (*P* = 0.001) and permanent (*P* = 0.001). Prior myocardial infarction (*P* = 0.005), percutaneous coronary intervention (*P* = 0.003) and coronary artery bypass grafting (*P* = 0.049) were more common, whereas coronary angiography during the index hospitalization was performed less frequently (*P* = 0.01) (Table 1). Moreover, acetylsalicylic acid, statins, loop diuretics, metformin and insulin were prescribed more often in patients with CHA_2_DS_2_-VASc ≥4 (Table 1).

### 3.2 Laboratory parameters

Patients with CHA_2_DS_2_-VASc ≥4 had lower baseline glomerular filtration rate (*P* < 0.001), potassium (*P* = 0.02), hemoglobin (*P* < 0.001) and hematocrit (*P* = 0.003) compared with those scoring < 4 (Table 2). Total cholesterol and triglycerides were also lower in the CHA_2_DS_2_-VASc ≥4 group (*P* = 0.04 and *P* = 0.03, respectively) (Table 2).

**Table 2:** Laboratory parameters in the studied groups.

Variable	CHA_2_DS_2_-VASc <4 (*n* = 48)	CHA_2_DS_2_-VASc ≥4 (*n* = 213)	*P*-value
NT-proBNP, pg/ml	1,680 (289–4,954)	2,109 (1,164–4,037)	0.30
Creatinine level on admission, mmol/L	101 (84–129)	101 (86–129)	0.98
eGFR on admission, ml/min/1.73 m^2^	75 (51–96)	55 (39–72)	**<0** **.** **001**
Maximal creatinine level, mmol/L	121 (85–142)	109 (93–139)	0.89
Sodium, mEq/L	140 (137–143)	141 (139–143)	0.25
Potassium, mEq/L	4.5 (4.3–4.8)	4.3 (4–4.7)	**0**.**023**
Hemoglobin, g/dl	13.6 (11.5–14.7)	12.2 (10.8–13.5)	**<0**.**001**
Hematocrit, %	40.7 (36.3–44.1)	37.4 (34.1–41.1)	**0**.**003**
MCV, fl	90.2 (87.3–93.3)	89.4 (84.6–93.4)	0.39
RDW, %	14.3 (13.5–16.9)	14.8 (13.8–16.8)	0.39
White blood cells, ×10^3 ^/µl	8.3 (6.2–10.7)	7.2 (6–9.1)	0.10
Platelet count, ×10^3 ^/µl	222 (183–254)	209 (174–259)	0.39
Glucose, mmol/L	6.1 (5.4–6.7)	6.1 (5.2–7)	0.82
Total cholesterol, mmol/L	4.4 (3.7–4.8)	3.9 (3.1–4.5)	**0**.**044**
LDL cholesterol, mmol/L	2.6 (2–3.2)	2.2 (1.7–2.7)	0.11
HDL cholesterol, mmol/L	1.1 (1–1.6)	1.2 (1–1.6)	0.56
Triglycerides, mmol/L	1.2 (0.9–1.9)	1 (0.8–1.4)	**0**.**036**

GFR, glomerular filtration rate; HDL, high-density lipoprotein; LDL, low-density lipoprotein; MCV, mean corpuscular volume; NT-proBNP, N-terminal pro-brain natriuretic peptide; RDW, red cell distribution width.

Bold value indicates *P* < 0.05.

### 3.3 Echocardiographic parameters

Echocardiography showed that patients with CHA_2_DS_2_-VASc ≥4 had smaller left-ventricular end-diastolic and end-systolic diameters, larger left-atrial diameter, and lower right-ventricular systolic pressure than those with scores <4 (Table 3). As expected, left-ventricular ejection fraction did not differ significantly between the groups (*P* = 0.55) (Table 3).

**Table 3:** Echocardiographic parameters in the studied groups.

Variable	CHA_2_DS_2_-VASc <4 (*n* = 48)	CHA_2_DS_2_-VASc ≥4 (*n* = 213)	*P*-value
LVEF at baseline, %	55 (55–60)	57 (50–60)	0.55
End-diastolic LV diameter, mm	51 (45–55)	48 (43–52)	**0** **.** **020**
End-systolic LV diameter, mm	37 (27–42)	31 (26–34)	**0**.**001**
Left atrium, mm	41 (37–51)	47 (43–52)	**0**.**008**
Left atrium area, cm^2^	26 (21–34)	29 (23–33)	0.64
E/A ratio	1.1 (0.7–1.8)	1.1 (0.7–2)	0.77
Right ventricular systolic pressure, mmHg	55 (40–63)	42 (34–55)	**0**.**015**
TAPSE, mm	20 (15–24)	19 (15–23)	0.99
Ascending aorta diameter, mm	35 (32–38)	36 (33–39)	0.26
Aortic valve peak gradient, mmHg	8 (6–18)	9 (6–22)	0.64

LVEF, left ventricular ejection fraction; TAPSE, tricuspid annular plane systolic excursion.

Bold value indicates *P* < 0.05.

### 3.4 Correlation analysis

Exploratory correlation analyses revealed that the CHA_2_DS_2_-VASc score correlated positively with age (*r* = 0.64, *P* < 0.001) and negatively with GFR (*r* = –0.35, *P* < 0.001). Age and GFR were inversely related (*r* = –0.52, *P* < 0.001), whereas left-ventricular end-diastolic and end-systolic diameters were positively correlated (*r* = 0.58, *P* < 0.001).

### 3.5 Long-term mortality

**Figure 2:**
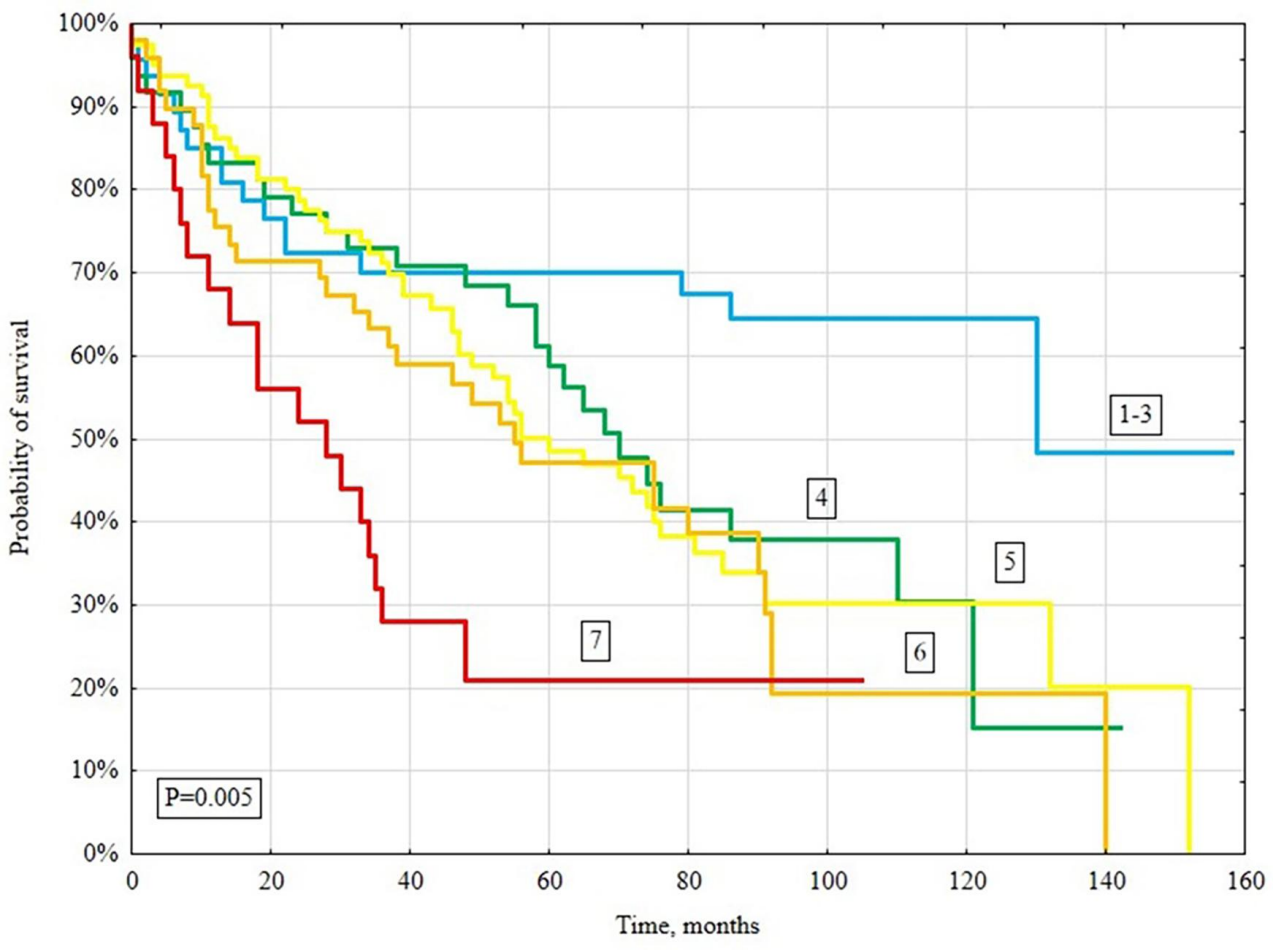
Kaplan–Meier survival curves for all-cause mortality based on the CHA_2_DS_2_-VASc score (1–3, 4, 5, 6, ≥7).

**Figure 3:**
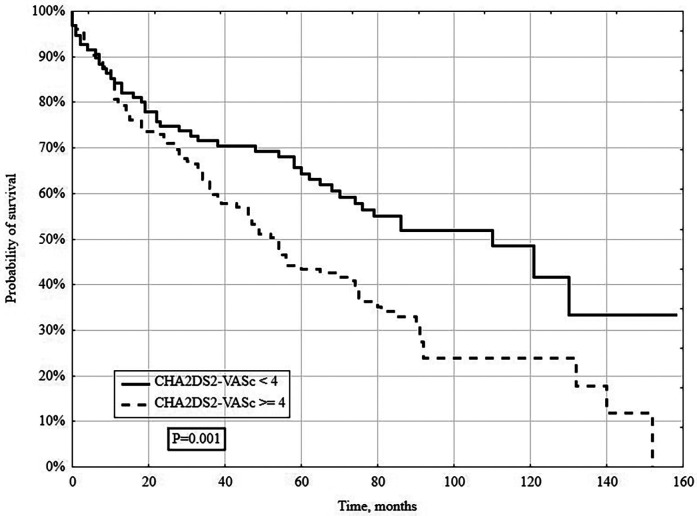
Kaplan–meier survival curve for all-cause mortality stratified by CHA_2_DS_2_-VASc <4 vs. ≥4.

In a long-term follow-up (median 4.3 years), 148/261 (56.7%) patients died. Deaths occurred in 131/213 (61.5%) patients with CHA_2_DS_2_-VASc ≥4 and 17/48 (35.4%) with CHA_2_DS_2_-VASc <4 (*P* = 0.005; Figure 2). When stratified by individual CHA_2_DS_2_-VASc categories (1–3, 4, 5, 6, ≥7), survival declined stepwise with higher scores (log-rank *P* = 0.005; Figure 2). Kaplan–Meier analysis showed significantly lower survival in the CHA_2_DS_2_-VASc ≥4 group (log-rank *P* = 0.001; Figure 3).

### 3.6 Multivariable cox regression analysis

In multivariable Cox regression, CHA_2_DS_2_-VASc dichotomised as ≥4 vs. <4 independently predicted all-cause mortality (*P* = 0.001), alongside NYHA class ≥III, COPD, lower hemoglobin and higher peak aortic-valve gradient (Table 4). When modeled as a continuous variable, each one-point increase in CHA_2_DS_2_-VASc was associated with a 32% higher risk of all-cause mortality (*P* = 0.001) (Table 5).

**Table 4:** Cox regression analysis of all-cause mortality prediction with CHA_2_DS_2_-VASc ≥4 (C-index 0.690; 95%: 0.639–0.741).

Variable	HR	95% CI	*P*-value
CHA_2_DS_2_-VASc ≥4	3.543	(1.677–7.486)	0.001
NYHA ≥3	1.994	(1.151–3.454)	0.014
COPD	1.945	(1.234–3.067)	0.004
Renal failure	1.414	(0.946–2.113)	0.091
Aortic valve peak gradient (per one mmHg)	1.009	(1.002–1.015)	0.01
Hemoglobin (per 1 unit)	0.834	(0.754–0.924)	<0.001

NYHA, New York Heart Association; COPD, chronic obstructive pulmonary disease; HR, hazard ratio; CI, confidence interval.

**Table 5:** Cox regression analysis of all-cause mortality prediction with CHA_2_DS_2_-VASc score expressed per one unit (C-index 0.691; 95% CI: 0.642–0.740).

Variable	HR	95% CI	*P*-value
NYHA ≥3	2.022	(1.17–3.496)	0.012
COPD	1.789	(1.134–2.822)	0.012
Renal failure	1.417	(0.951–2.111)	0.087
CHA_2_DS_2_-VASc (per one unit)	1.322	(1.126–1.552)	0.001
Aortic valve peak gradient (per one mmHg)	1.007	(1.001–1.014)	0.023
Hemoglobin (per 1 unit)	0.827	(0.747–0.915)	<0.001

NYHA, New York Heart Association; COPD, chronic obstructive pulmonary disease; HR, hazard ratio; CI, confidence interval.

### 3.7 Comparison with other scales–ROC analyses

**Figure 4:**
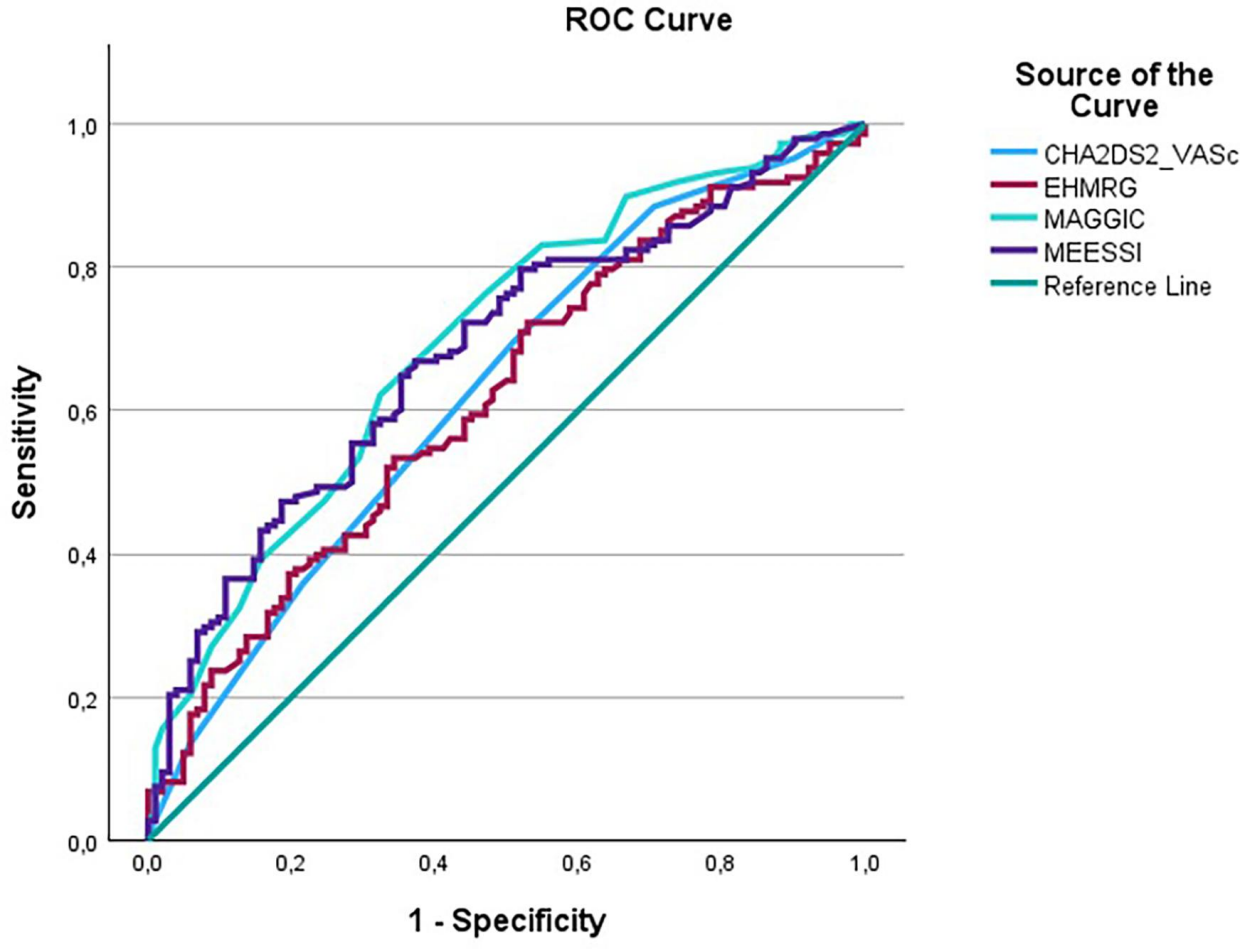
Overlaid ROC curves comparing discrimination across CHA_2_DS_2_-VASc, MAGGIC, EHMRG and MEESSI-AHF for all-cause mortality.

In head-to-head ROC analyses for all-cause mortality, the area under the curve (AUC) was 0.629 (95% CI 0.559–0.699) for CHA_2_DS_2_-VASc, 0.697 (95% CI 0.631–0.763) for MAGGIC, 0.618 (95% CI 0.548–0.688) for EHMRG, and 0.682 (95% CI 0.616–0.748) for MEESSI-AHF. In pairwise AUC comparisons there was no statistically significant difference between the scores (all *P* > 0.05) and each score discriminated mortality significantly above chance (AUC > 0.5; all *P* < 0.01). The corresponding overlaid ROC curve is shown in Figure 4; individual curves are provided in Supplementary Figures S1a–d.

## 4 Discussion

### 4.1 Principal findings

To our knowledge, this study is the first to demonstrate that the CHA_2_DS_2_-VASc score independently predicts long-term mortality in patients hospitalized for acute decompensated HFpEF, irrespective of atrial fibrillation. Each one-point increase in the score was associated with a 32% higher risk of death.

These data suggest that a score originally devised for thromboembolic risk in atrial fibrillation can capture global vulnerability in HFpEF, where multimorbidity and systemic dysfunction drive outcomes.

### 4.2 Context within existing risk tools

Risk stratification in HFpEF remains challenging. Although tools like MAGGIC, EHMRG and MEESSI-AHF are validated, they require numerous inputs and often focus on short-term horizons (7–30 days), which limits bedside uptake in routine wards and at discharge planning in HFpEF patients (13, 14, 37). In contrast, CHA_2_DS_2_-VASc uses information universally collected on admission and clinic visits (age, sex, hypertension, diabetes, vascular disease, stroke history, HF), enabling immediate calculation without calculators or laboratory dependencies (16). The simplicity of a single integer that stratifies long-term risk is especially attractive in HFpEF, a syndrome with high readmission and mortality risk but limited disease-modifying therapies compared with HFrEF (10, 11, 27).

In this cohort, discrimination across CHA_2_DS_2_-VASc, MAGGIC, EHMRG, and MEESSI-AHF was broadly similar, with no statistically significant differences in AUC on pairwise testing. Accordingly, the practical advantage of CHA₂DS₂-VASc is its simplicity and universal availability at admission, rather than superior discrimination.

### 4.3 Alignment and divergence from prior literature

Our findings extend prior observations showing that CHA₂DS₂-VASc predicts adverse outcomes beyond AF. The score has shown prognostic value across chronic kidney disease, COPD, and acute myocardial infarction cohorts, indicating it functions as a marker of aggregated cardiovascular risk rather than a purely embolic tool (17, 19, 20). In heart failure specifically, the score predicted mortality across LVEF phenotypes in large cohorts and registries, including HFrEF and mixed HF populations, supporting its generalisability (23, 25, 26). By contrast, a *post-hoc* analysis of TOPCAT did not demonstrate independent associations between CHA_2_DS_2_-VASc and outcomes in a mixed acute/chronic HFpEF cohort with LVEF ≥ 45%, a difference likely related to population composition, event rates, and endpoint structure (12). Importantly, our cohort comprised exclusively acutely decompensated HFpEF—patients in whom pragmatic, fast triage tools are most needed at the point of care (10, 14, 37).

### 4.4 Clinical differences by CHA_2_Ds_2_-VASc category

In the present cohort, patients with CHA_2_DS_2_-VASc ≥4 differed substantially from those with CHA_2_DS_2_-VASc <4. Multimorbidity clustered within the higher-score group and, as expected, was associated with increasing CHA_2_DS_2_-VASc values. A cut-off of 4 points has been commonly used in previous studies and effectively identifies patients at higher risk of ischaemic stroke and other thromboembolic events compared with lower scores (38). Moreover, higher CHA_2_DS_2_-VASc values have been associated with increased mortality and adverse cardiovascular outcomes in populations with atrial fibrillation and heart failure, supporting its broader prognostic signal beyond embolic risk alone (39).

### 4.5 Medication patterns and procedural context

In our population, increasing CHA_2_DS_2_-VASc scores were accompanied by more frequent use of loop diuretics, which likely reflects greater clinical severity at decompensation. Consistent with a higher comorbidity burden, patients with CHA_2_DS_2_-VASc ≥4 also more often received statins, acetylsalicylic acid, metformin and insulin, aligning with prior observations that polypharmacy in heart-failure care tracks with cardiometabolic multimorbidity (40, 41). Interestingly, coronary angiography during the index hospitalization was performed less often in the high-score group. This may reflect prior angiographic evaluation and previous percutaneous coronary interventions, as well as clinical judgement that prioritized medical management in patients with advanced age and comorbidity.

### 4.6 Echocardiographic phenotype and structural remodelling

We observed that end-diastolic and end-systolic LV diameters were larger in patients with CHA_2_DS_2_-VASc <4 than in those with scores ≥4, whereas left-atrial size was greater in the higher-score group. This pattern is compatible with a phenotype of long-standing hypertension and concentric remodelling in patients with higher CHA_2_DS_2_-VASc values, who frequently accumulate vascular risk factors over time (42, 43). Given that components of the CHA_2_DS_2_-VASc score are established risk factors for composite cardiovascular outcomes, this likely explains why the score stratifies all-cause mortality in HFpEF irrespective of atrial fibrillation (44, 45).

### 4.7 Thromboembolic risk across heart-failure phenotypes

It is worth taking into account that thromboembolic complications are associated with all subtypes of HF (46–48). The reasons for this phenomenon might be due to increased concentrations of prothrombotic molecules, systemic inflammation, endothelial dysfunction and disruption of the Virchow triad regardless of AF (46–48). Notably, patients with HFpEF are more often characterized by comorbidities indirectly increasing thromboembolic risk than in other HF subtypes, especially with coexisting AF (46–48). Given that comorbidities included in the CHA_2_DS_2_-VASc scale increase thromboembolic complications it might be useful to estimate the thromboembolic risk in HF and sinus rhythm (46–48).

### 4.8 Strengths and limitations in interpretation

Strengths include a consecutive real-world cohort, linkage to a national death registry, and consistent HFpEF definition according to contemporaneous ESC guidance across the study window (30–33). This study has some limitations. First, it is a single-centre registry with a relatively small HFpEF cohort and a limited number of patients with a CHA_2_DS_2_-VASc score <4 (*n* = 48). This modest sample size reflects the low incidence of HFpEF admissions to a tertiary centre that mainly treats advanced HFrEF. However, it reduces the statistical power of subgroup analyses and leads to wide confidence intervals in the multivariable models, so our estimates should be viewed as exploratory. Second, as we focused on all-cause mortality, future studies should assess other cardiovascular endpoints and the impact of evolving pharmacotherapies over longer follow-up. Third, because recruitment spanned 2009–2022, we could not evaluate the influence of sodium–glucose co-transport-2 inhibitors or other recent guideline-directed therapies on HFpEF outcomes. Taken together, these factors warrant cautious interpretation of our findings and highlight the need for validation in larger, multicentre cohorts.

## 5 Conclusions

In the current study we indicated that patients with acute decompensated HFpEF are characterized by a higher all-cause mortality risk with each incremental point in the CHA_2_DS_2_-VASc score, regardless of the presence of AF. Given its predictive value for mortality, the CHA_2_DS_2_-VASc score may serve as a simple and practical tool for risk stratification in patients with HFpEF, identifying those at higher risk who may benefit from more aggressive monitoring and therapeutic strategies in a syndrome that remains heterogeneous and difficult to treat. Future multicenter studies are required to confirm our observations.

## Data Availability

The original contributions presented in the study are included in the article/Supplementary Material, further inquiries can be directed to the corresponding author.

## Supplementary material

Supplementary Figure S1 Receiver operating characteristic (ROC) curves for prediction of all-cause mortality: **(a)** CHA_2_DS_2_-VASc; **(b)** MAGGIC; **(c)** EHMRG; **(d)** MEESSI-AHF.
