# Supplementary figures and images for: CHA2DS2-VASc score as a mortality predictor in acute heart failure with preserved ejection fraction
Source: Front Cardiovasc Med. 2025 Nov 3;12:1611825. doi: 10.3389/fcvm.2025.1611825 (PMC12620384; doi:10.3389/fcvm.2025.1611825)

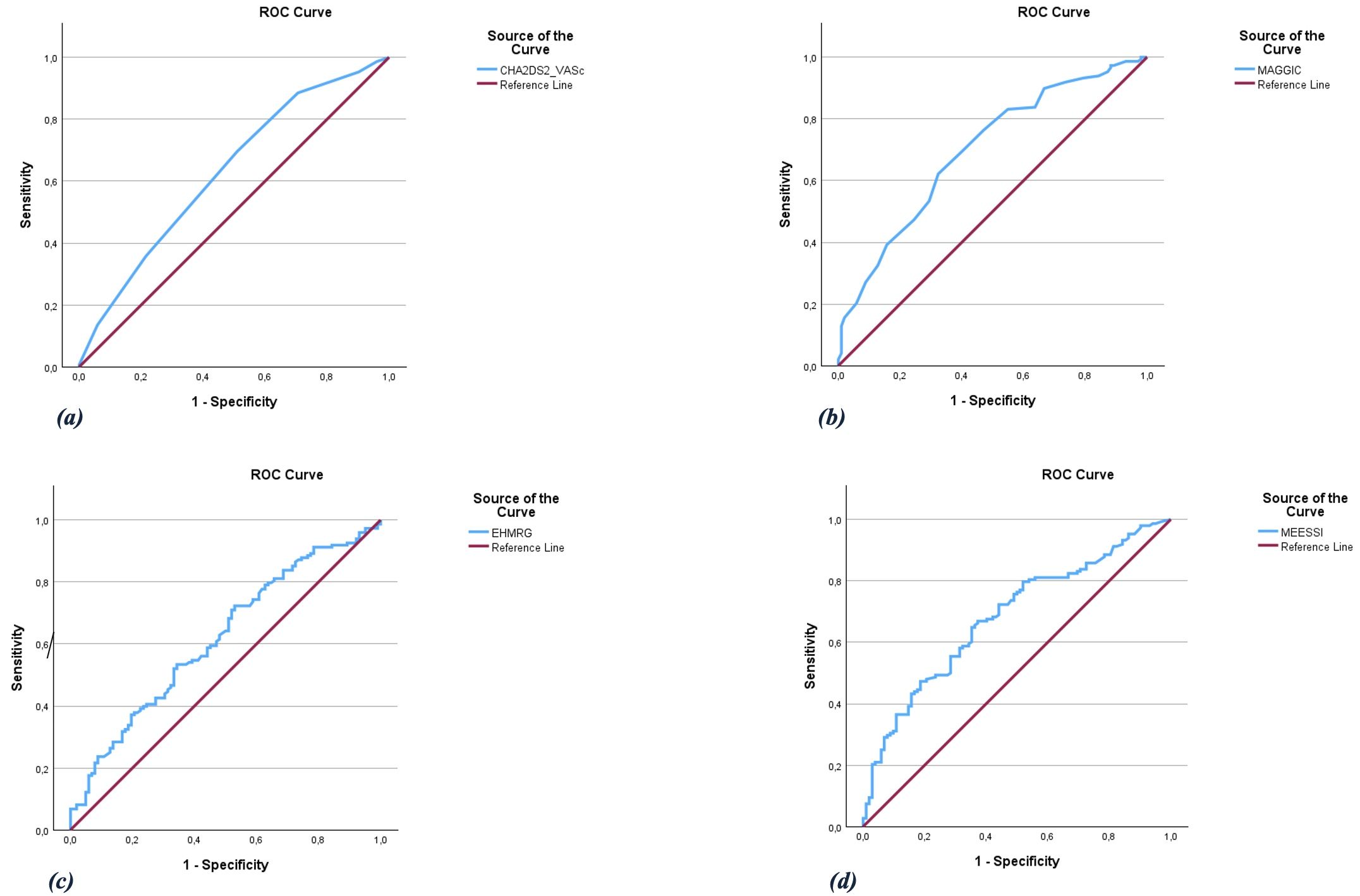

Supplement: Supplementary Figure S1 — Receiver operating characteristic (ROC) curves for prediction of all-cause mortality: (a) CHA2DS2-VASc; (b) MAGGIC; (c) EHMRG; (d) MEESSI-AHF. [file Image1.jpeg]
